# Surgical Emergencies

**Published:** 1875-03

**Authors:** 


*Surgical Emergencies: Together with the Emergencies attendant on Parturition and the Treatment of Poisoning.* By William P. Swain, F. R. C. S. With eighty-two illustrations. Philadelphia: Lindsay & Blakiston, 1874.

This little book is a compilation from the best surgical authorities of the best methods of procedure in the ordinary surgical emergencies. It treats of injuries of the head, eye, mouth and adjacent parts, the abdominal thoracic, and pelvic regions, and of the extremities. A chapter each is devoted to the subject of parturition, poisoning and antiseptic treatment; the latter being an account of the method of Prof. Lister. The concluding chapter is upon apparatus and dressings, and gives a brief general account of what may be extemporized in emergencies.

As a whole the work is one of considerable value and will be found to be a convenient book of reference, as well as instructive for general reading.

